# The Report of Lightning in Himalayan Locale

**DOI:** 10.1155/2023/1888382

**Published:** 2023-06-22

**Authors:** Pitri Bhakta Adhikari

**Affiliations:** Tri-Chandra Multiple Campus, Tribhuvan University, Kathmandu, Nepal

## Abstract

A few normal calamities (disasters) as often as possible happen within the Himalayan locale in Nepal. The height of this locale ranges from 59 m to “8848.86 m” along the range of 160 km. As a result, there is a significant variety of temperatures within the locale. In addition, Nepal includes a heterogeneous geography. All these highlights impact different normal fiascos counting the lightning action. This report points at analyzing the varieties of lightning inside and over a long time from January 2011 to present. For this report, the information was taken from the Disaster Risk Reduction (DRR) portal of the Ministry of Home Affairs (MOHA). The investigation indicated that there was no lightning occasion in November, and the lightning stroke density was higher in the premonsoon period, and the number of harmed individuals was almost three times the number of individuals passing due to the lightning.

## 1. Introduction

The nation of the Mount Everest, Nepal, lies on the north side of the equator of scope 26.37°N to 30.45°N and longitude 80.066°E to 88.2°E in a Himalayan locale. In the Himalayan regions, there are different cloud structures [1–3], and the lightning and its effects are explained in [4–7]. The height of the nation ranges heterogeneously from 59 m to 8848.86 m. The arrival of the least elevation which of the most elevated height (Mount Everest) lies inside the range of 160 km, and the temperature difference is around 95°C. Due to the variety of temperatures in a short range, there are differing qualities of climate and the variety of climate marvels [8]. Water actually streams from tall elevation to low elevation with high speed due to the tremendous contrast of height in a short range. This leads to the occurrence of significant disasters which comes about the misfortune of human lives and cattle with the annihilation of physical properties of billions of dollars [9]. Other than human casualties, the death of cattle was also reported and modern parts of electronic, military, and restorative medical equipment can be destroyed. Besides this, the communication and transmission lines are affected by radiation produced due to lightning.

Gomes et al. [10] clarified that the passing or harm of the individuals depends upon the different variables. These variables may be the distance of the lightning, step potential, current magnitudes, temperature during lightning, and so on. They also detailed that the lightning causes harm to the human creatures and household creatures, when they are in open land-fields, and do not take shelter under the tall trees during the lightning. Gomes [11] detailed that lightning is taken as a calamity since the topographical situation within the context of Sri Lanka within the hilly locale and casualty of individual people are not detailed precisely due to the scattered data on the Himalayan locale. Baral and Mackerras [12] detailed that more positive lightning occurs within the slope hill and precipitous mountainous locale. Uman [13] and Rakov and Uman [14] clarified that the marvels happen due to high current 300 kA and high temperature up to 30,000 K.

## 2. Methodology

In this research report, the data were taken from the Disaster Risk Reduction (DRR) portal of the Ministry of Home Affairs (MOHA) from January, 2011, to December, 2021, and were analyzed in terms of their inter- and intra-annual variations and their distribution over the hill and mountainous locale in Nepal.

## 3. Observation and Discussion

**Figure 1:**
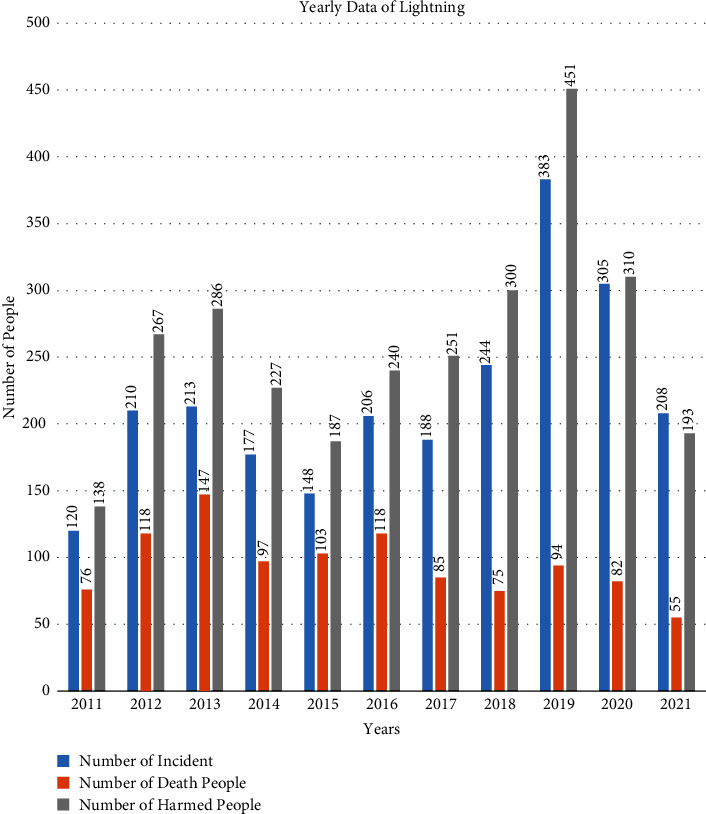
The bar diagram of the annual distribution of lightning.

Nepal has one of the most elevated chances of disaster due to its topographical structure. The northern upper portion of Nepal incorporates the rough hilly district secured by the tall Himalayas, the lower southern portion comprises plain Terai, and the uneven district lies in between the two. Floods, landslides, avalanches, thunderbolts, electrical storms, and fires happen regularly as a disaster [9]. Within the Himalayan district, more than 250 creatures were killed due to a single stroke of the lightning [15]. The phenomenon of lightning in this geological structure is exceptionally critical. As specified prior, the current and temperature are very significant factors for the process of lightning. The death of human creature due to lightning in Nepal is presented in Figure 1.

The number of occurrences and number of passing and harmed individuals due to lightning in twelve months were observed and analyzed here and are presented in Table 1.

**Figure 2:**
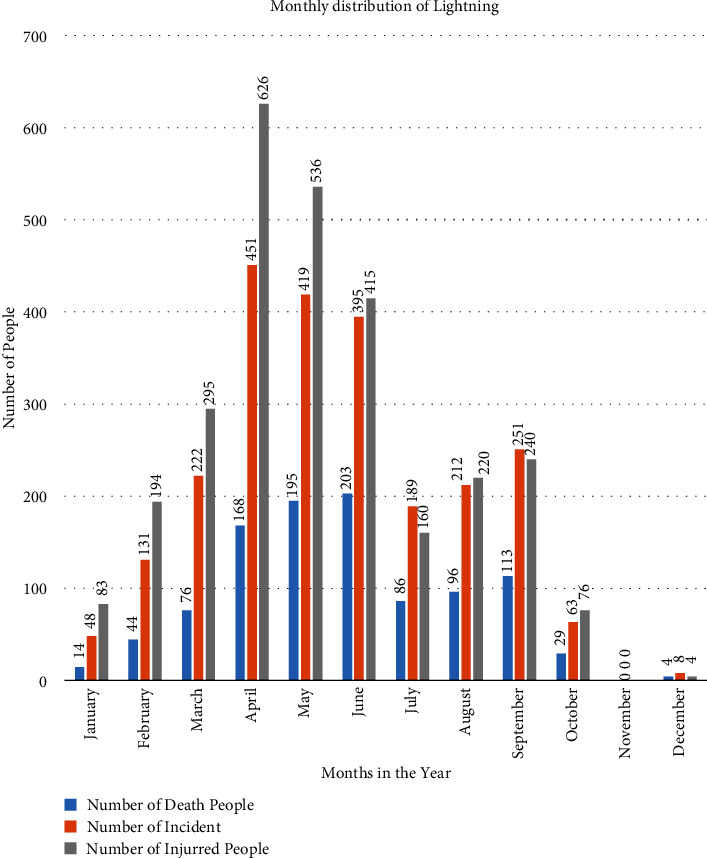
The bar diagram of the monthly distribution of the lightning during this period.

The bar graph of the month-to-month distribution of lightning during this era is shown in Figure 2. In Figure 2, the number of incidents and the number of passing and harmed individuals because of lightning occasions communicated month to month and these conveyances of lightning were observed and analyzed. There are no lightning occasions in November, and therefore, the maximum number of lightning incidents happens in the premonsoon period. In addition, the number of harmed individuals is almost 3 times above the number of passing individuals. The seventy-seven districts are taken into account as a sample area for the distribution of the lightning occasions. The information available on the DRR portal of seventy-seven districts in Nepal was analyzed by using ArcGIS software.

**Figure 3:**
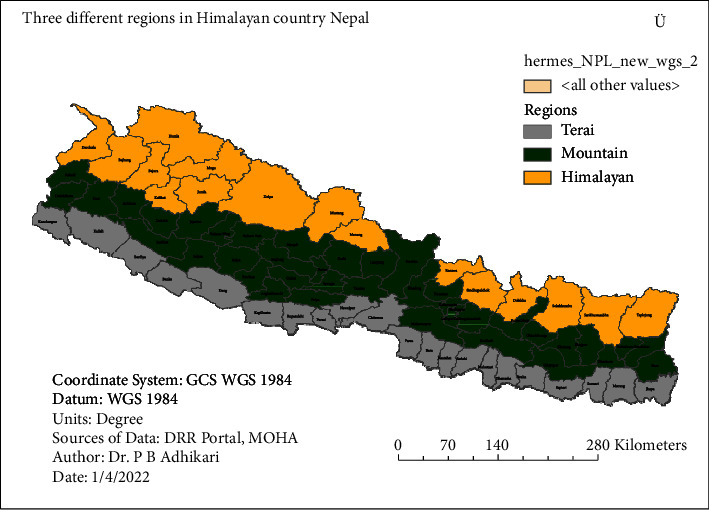
Three different locales, Terai, mountain, and Himalayan, the investigated zone of Nepal.

The investigated zone is categorized into three locales, namely, Terai, mountain, and Himalayan. Terai lies beneath 600 m from the ocean level and the high Himalayan locale lies at an altitude of over 5000 m. The center part between them is called hilly or mountainous locale, which is the highly affected area because of lightning as shown in Figure 3. The fatality and harmed individual rate over this area are significantly high because of the higher population density and lightning flash density.

**Figure 4:**
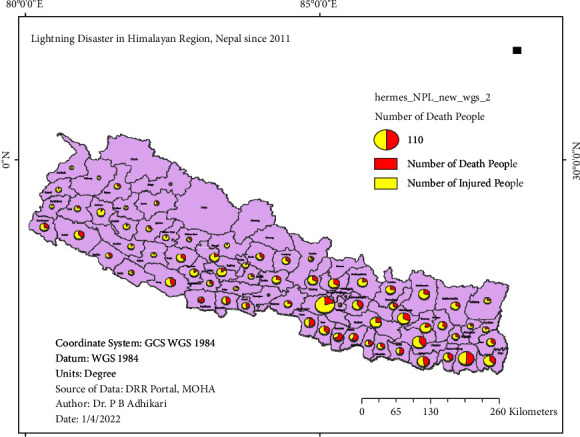
The proportionate presentation of fatalities and injured individuals. The size of each pie chart corresponds to the number of lightning incidents.

**Figure 5:**
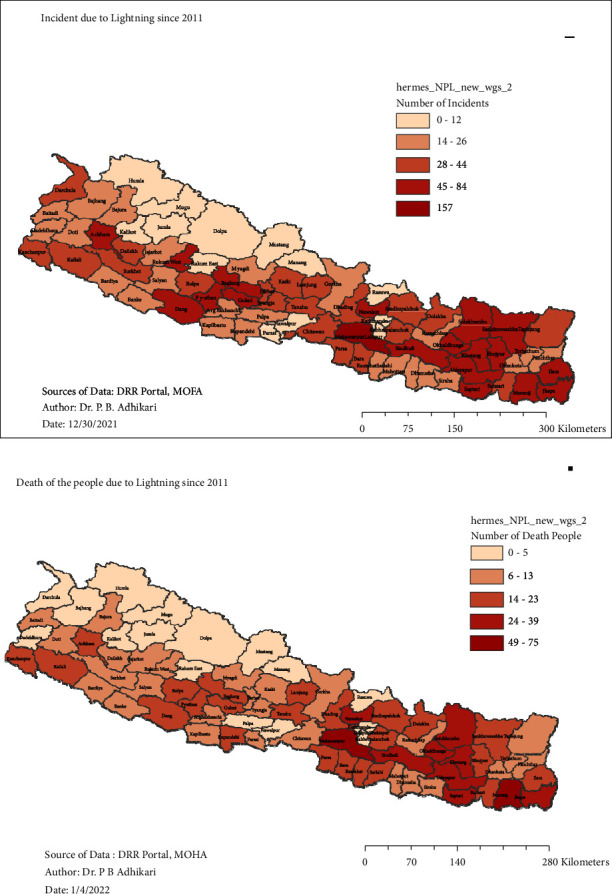
The occurrence of lightning incidents (a) and the number of deaths resulting from these incidents (b).

To determine the effect of electrical storms, the occurrences of lightning incident events, death of the people, and harmed individuals due to lightning were observed and analyzed. By utilizing the software program ArcMap, the presentation of passed individuals and harmed people was analyzed. The occurrence of lightning events is shown in the pie diagram inside the map of Nepal, and the size of the pie diagram varies with the number of lightning events, as shown in Figure 4. Similarly, the incident of the lightning events and also the death of the people within the seventy-seven districts are presented in Figure 5 by using the software program of ArcGIS mapping.

**Figure 6:**
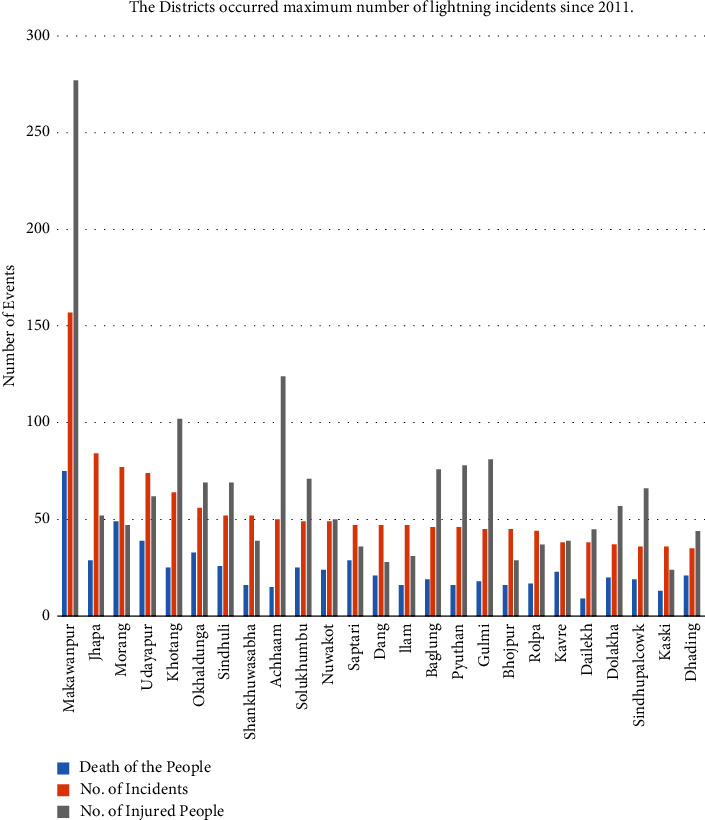
District-wise maximum number of lightning incidents during this period.

**Figure 7:**
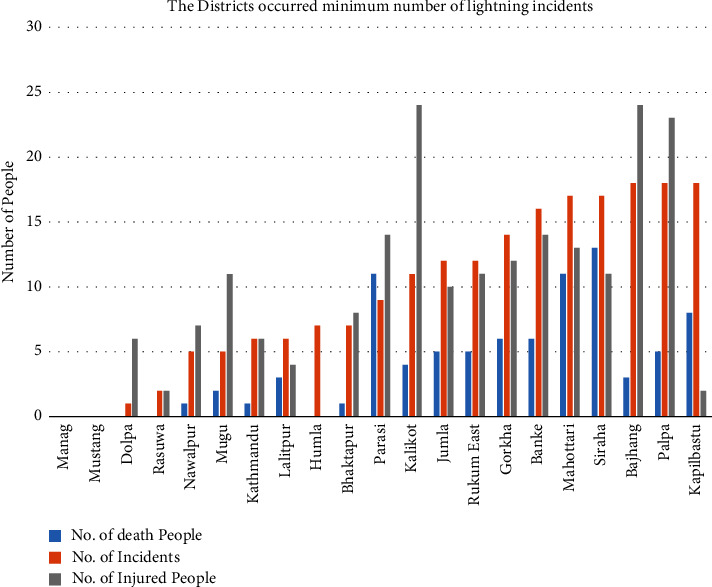
District-wise minimum number of lightning incidents during this period.

Again, among all the 77 districts of Nepal, only the lightning occasion happened in the most extreme 25 districts which are displayed in the chart in Figure 6. During this period, Makawanpur district showed the greatest harm caused, followed by Jhapa district. But in contrast, in the same time period, there were no lightning occurrences in Manang and Mustang districts according to the DRR portal. The minimum number of lightning incidents due to the low population density at the place of tall elevation is shown in Figure 7.

## 4. Results and Discussion

**Figure 8:**
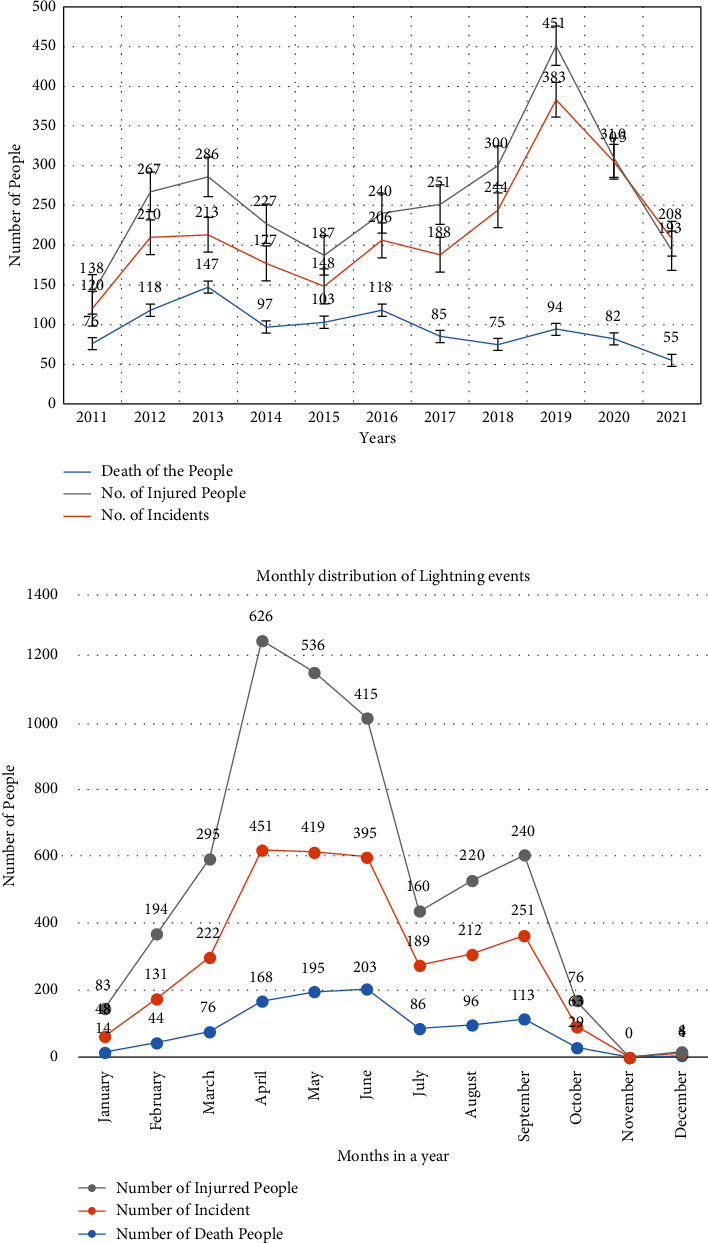
The annual (a) and monthly distribution (b) of the lightning incidents.

On the basis of casualty of the people passing and harmed, loss of cattle, causing fires in gigantic wilderness, and unwittingly harming TVs, computers, radios, phones, fridges, electronics gazettes, various equipment, medical equipment, causing fires in buildings due to high voltage, etc., the lightning can be taken as one of the major disasters. The distribution of the lightning incidents showed that the number of harmed people is thrice that of the number of passed people and the harmed people are as high in the premonsoon period as the dead people.  Figure 8(a) represents the annual distribution of lightning and Figure 8(b) represents the monthly distribution of lightning. There are no lightning incidents in the month of November, and the maximum number of lightning incidents occurs during the premonsoon period. During the premonsoon period of April, May, and June, the maximum number of lightning incidents occurred and casualties were also high in the same period, as shown in Figure 8.

## 5. Conclusion

Lightning is the main disaster in hilly locales due to the topographical features. It influences the environment of this region, and different temperatures occur in the short range. The month-to-month and annual distributions of lightning were observed and analyzed. There are no lightning incidents in the month of November during the research period, and the maximum number of lightning incidents occurs during the premonsoon period. It occurred in the months of April, May, and June in the premonsoon period, and the casualties were also high in the same period. The distribution of the lightning phenomena in seventy-seven districts of Nepal is observed and analyzed on the basis of the data available on the DRR portal. To be safe from lightning disasters, the research on lightning activity is very essential that really helps minimize the risk of disaster. Hence, it is recommended to the concerned authority to conduct an awareness program for the various people such as school children, population of mountainous regions, farmers, local people, and local government to diminish the risk of lightning.

## Figures and Tables

**Table 1:** The monthly distribution of lightning of the latest eleven years.

Year	No. of events	2011	2012	2013	2014	2015	2016	2017	2018	2019	2020	2021	Total
January	Incident	—	3	7	1	2	—	—	0	23	11	1	48
	Death	—	2	5	0	1	—	—	0	5	1	0	14
	Harmed	—	10	16	1	2	—	—	0	46	8	0	83

February	Incident	—	9	14	6	21	2	0	4	47	18	10	131
	Death	—	2	13	0	10	2	0	0	9	6	2	44
	Harmed	—	27	15	15	37	—	0	24	57	8	11	194

March	Incident	—	6	26	12	24	15	23	32	21	45	18	222
	Death	—	3	20	3	14	10	2	14	2	4	4	76
	Harmed	—	18	74	15	13	18	33	31	29	44	20	295

April	Incident	11	51	51	17	21	13	37	62	56	69	63	451
	Death	5	22	25	7	17	5	21	24	15	14	13	168
	Harmed	9	102	80	25	22	26	45	66	110	100	41	626

May	Incident	23	30	17	40	27	50	65	32	51	46	38	419
	Death	15	27	12	26	21	26	25	11	9	15	8	195
	Harmed	31	50	23	45	54	43	96	46	56	45	47	536

June	Incident	19	21	37	48	25	51	19	58	54	32	31	395
	Death	14	20	28	28	22	32	10	17	24	17	13	203
	Harmed	19	21	28	64	18	68	24	72	45	27	29	415

July	Incident	22	26	14	9	10	2	16	19	40	23	8	189
	Death	13	12	11	4	5	2	11	6	11	7	4	86
	Harmed	22	10	8	19	5	6	17	14	31	26	2	160

August	Incident	9	30	25	9	6	40	12	3	50	23	5	212
	Death	5	16	18	4	5	17	7	0	13	7	4	96
	Harmed	19	12	24	3	13	43	21	8	47	24	6	220

September	Incident	25	19	19	25	3	25	11	30	29	35	30	251
	Death	17	12	13	18	2	18	9	3	4	11	6	113
	Harmed	21	17	16	34	—	31	8	31	26	23	33	240

October	Incident	11	2	2	5	9	8	5	2	12	3	4	63
	Death	7	2	1	4	6	6	0	0	2	0	1	29
	Harmed	17	—	2	4	23	5	7	5	4	5	4	76

November	Incident	—	—	—	—	—	—	—	—	—	—	—	—
	Death	—	—	—	—	—	—	—	—	—	—	—	—
	Harmed	—	—	—	—	—	—	—	—	—	—	—	—

December	Incident	—	—	1	5	—	—	—	2	—	—	—	8
	Death	—	—	1	3	—	—	—	0	—	—	—	4
	Harmed	—	—	—	1	—	—	—	3	—	—	—	4

Total	Incident	120	210	213	177	148	206	188	244	383	305	208	2402
	Death	76	118	147	97	103	118	85	75	94	82	55	1050
	Harmed	138	267	286	227	187	240	251	300	451	310	193	2850

## Data Availability

The data can be obtained from the corresponding author upon reasonable request.

## References

[1] Williams E. (1989). The tri-pole structure of thunderstorms. *Journal of Geophysical Research*.

[2] Krehbiel P. R. (1986). *The Electrical Structure of Thunderstorm, the Earth’s Electrical Environment*.

[3] MacGorman D. R., Rust W. D. (1998). *The Electrical Nature of Storms*.

[4] Barros A., Lang T. (2003). Monitoring the monsoon in the Himalayas: observations in Central Nepal, June 2001. *Monthly Weather Review*.

[5] Berger K. (1977). The earth flash. *Lightning, Physics of Lightning*.

[6] Cooray V. (2015). *An Introduction to Lightning*.

[7] Malan D. J. (1963). *Physics of Lightning*.

[8] Wu T., Wang D., Takagi N. (2019). Intracloud lightning flashes initiated at high altitudes and dominated by downward positive leaders. *Journal of Geophysical Research: Atmospheres*.

[9] Nepal Disaster Report (2019). *National Emergency Operation Center*.

[10] Gomes C., Ahmed M., Hussain F., Abeysinghe K. R. Lightning Accidents and Awareness in South Asia: Experience in Sri Lanka and Bangladesh.

[11] Gomes C. (2013). *Lightning, Hazard Profiles of Sri Lanka*.

[12] Baral K., Mackerras D. (1993). Positive cloud-to-ground lightning discharges in Kathmandu Thunderstorms. *Journal of Geophysical Research*.

[13] Uman M. A. (2001). *The Lightning Discharge*.

[14] Rakov V. A., Uman M. A. (2003). *Lightning: Physics and Effects*.

[15] Adhikari P. B., Adhikari A., Tiwari A. K. (2021). Effects of lightning as a disaster in Himalayan region. *Bibechana*.

[16] Albrecht R. I., Goodman S. J., Buechler D. E., Blakeslee R. J., Christian H. J. (2016). Where are the lightning hotspots on earth? Bulletin of the American meteorological society.

[17] Biswas A., Dalal K., Hossain J., Baset K. U., Rahman F., Mashreky S. R. (2016). Lightning injury is a disaster in Bangladesh? exploring its magnitude and public health needs. *F1000Research*.

[18] Dewan A., Hossain M. F., Rahman M. M., Yamane Y., Holle R. L. (2017). Recent lightning-related fatalities and injuries in Bangladesh. Weather. *Climate, and Society*.

[19] Mäkelä A., Shrestha R., Karki R. (2014). Thunderstorm characteristics in Nepal during the pre-monsoon season 2012. *Atmospheric Research*.

